# Practical Points

**Published:** 1894-12-01

**Authors:** 


### PRACTICAL POINTS.

*Hospitals and Advertising.—A correspondent has had his attention drawn to a consideration which he suggests may sometimes not be very forcibly present to the minds of those who criticise, he trusts in no unfriendly spirit, the "management expenses" of the smaller hospitals. He points out (for the sake of argument) that, supposing a large hospital expends, say,* £20 *in whole page advertisements in "annual" publications, and its management expenses are* £2,000, *such expenditure only forms one-hundredth part of the management expenditure; while in smaller hospitals, expending only* £10 *in, say, half page advertisements and* £300 *on management, the expenditure on advertisements forms one-thirtieth of the larger amount. And yet he remarks the publicity obtained is, if anything, less.*

This correspondent overlooks the fact that an institution with an expenditure of a few hundreds a year ought to be able easily to raise the necessary income from the special class of persons whom it particularly benefits. The larger institution has to appeal to a much wider area, because its work benefits all classes to a sufficient extent to warrant such an appeal. Hence advertising is not usually necessary, or even of service, to the smaller institution, save only so far as it is directed to keep the institution under the eye of those who are likely to make bequests. This publicity can be obtained for a very small expenditure by selecting one or more annuals which circulate amongst these classes, to whom they frequently become books of permanent reference.

